# Crust‒mantle interaction and fluid evolution in the Arabian‒Nubian Shield: evidence from Abu Aqarib A-type granites, Eastern Desert of Egypt

**DOI:** 10.1038/s41598-026-65694-z

**Published:** 2026-08-18

**Authors:** Shaimaa A. El-Shafei, Mokhles K. Azer, Abdel-Aal M. Abdel-Karim, Waheed I. Elwan

**Affiliations:** 1 Geology Department, Faculty of Science, Zagazig University, Zagazig, 44519 Egypt; 2 Geological Sciences Department, National Research Centre, Cairo, Egypt

**Keywords:** Abu Aqarib, Hypersolvus granites, Episyenite, Petrogenesis, Metasomatism, Planetary science, Solid Earth sciences

## Abstract

Post-collisional A-type granites provide important insights into crust–mantle interaction and late-stage hydrothermal activity in the Arabian–Nubian Shield. This study integrates petrography, mineral chemistry, and whole-rock geochemistry to investigate Abu Aqarib alkali feldspar granites in the Eastern Desert of Egypt. The aim is to evaluate magma sources, magmatic evolution, tectonic setting, and fluid-driven alteration. The granites are ferroan hypersolvus A-type varieties characterized by high SiO₂ (up to 76.5 wt%), elevated total alkalis (up to 8.6 wt%), enrichment in light rare-earth elements (LREEs), and negative Eu anomalies. Zircon saturation thermometry indicates high crystallization temperatures (875–1018 °C), whereas pressure estimates suggest shallow crustal emplacement (< 6.5 km) under reduced conditions (log ƒO₂ ≈ − 10). Geochemical signatures indicate derivation predominantly from partial melting of metasedimentary crustal sources, with limited mantle involvement during post-collisional extension. The generated magma subsequently evolved through extensive fractional crystallization, with possible minor crustal assimilation during its ascent. Petrographic observations and isocon mass-balance analyses indicate that late-stage fluid-rock interaction led to episyenitization. This process is marked by alkali metasomatism, quartz dissolution, and redistribution of several rare-metal and trace elements, including Nb, Ta, Th, U, Y, and heavy REEs (HREEs). A conceptual model is proposed in which evolved A-type magmatism was followed by structurally controlled fluid circulation and hydrothermal alteration. The coupled effects of magmatic differentiation and fluid–rock interaction contributed to rare-metal redistribution in Abu Aqarib AFGs. These findings offer novel insights into the processes controlling rare-metal concentration in post-collisional A-type granites and contribute to a broader understanding of post-collisional magmatic–hydrothermal evolution in similar granite provinces worldwide.

**Supplementary Information:**

The online version contains supplementary material available at 10.1038/s41598-026-65694-z.

## Introduction

The basement rocks in the Eastern Desert (ED) of Egypt form the northeastern part of the Neoproterozoic Arabian-Nubian Shield (ANS). This shield represents a juvenile continental crust formed by the accretion of oceanic and continental magmatic arcs^1^. Accretion took place during the closure of the Mozambique Ocean and the subsequent collision between West and East Gondwana^2,3^. The terminal stage of ANS evolution was dominated by widespread post-collisional magmatism, recording the transition from compressional to extensional tectonic regimes. During this stage, voluminous suites of high-K calc-alkaline and alkaline granitoids were emplaced. These intrusions developed after continental collision and continued until the onset of crustal extension and within-plate rifting^4–9^.

Granitoids constitute ~ 50% of the exposed intrusive rocks in the Egyptian basement complex. They are compositionally and chronologically classified into four main suites: (1) syn-collisional calc-alkaline granitoids (∼870–750 Ma), (2) late- to post-collisional calc-alkaline granitoids (∼710–650 Ma), (3) post-collisional alkaline A-type granites (∼630–580 Ma), and (4) anorogenic alkaline/peralkaline granites (∼590–540 Ma)^6,9,10^. Post-collisional alkaline A-type granites play an important role in the stabilization of ANS crust^11^. Their distinctive petrological and geochemical characteristics provide key insights into crust–mantle interaction and continental crust evolution. In addition, these granites have recently gained considerable attention owing to their potential economic importance as sources of rare earth elements (REEs), uranium (U), thorium (Th), tantalum (Ta), and niobium (Nb).

Despite numerous studies on post-collisional A-type granites in ED, their petrogenesis, source characteristics, and the role of late-stage fluids remain debated, particularly in poorly studied intrusions. In this context, the Abu Aqarib alkali feldspar granites (AFGs) in the central Eastern Desert (CED) provide an ideal case study to investigate the origin and evolution of post-collisional A-type magmatism in the ANS.

Previous literature on Abu Aqarib AFGs is limited, focusing mainly on field observations and petrography^12,13^ or on radioactive mineralization using remote sensing approaches^14,15^. A comprehensive petrogenetic model integrating magmatic evolution and post-magmatic alteration is still lacking. Therefore, the present study integrates field observations, petrography, mineral chemistry, and whole-rock geochemistry to constrain the petrogenesis and tectono-magmatic evolution of Abu Aqarib AFGs. It also evaluates the role of hydrothermal fluids in modifying their mineralogical and geochemical characteristics. Particular attention is given to fluid-driven alteration and its implications for rare-metal enrichment and the late crustal evolution of the ANS.

## Geologic setting

**Fig. 1:**
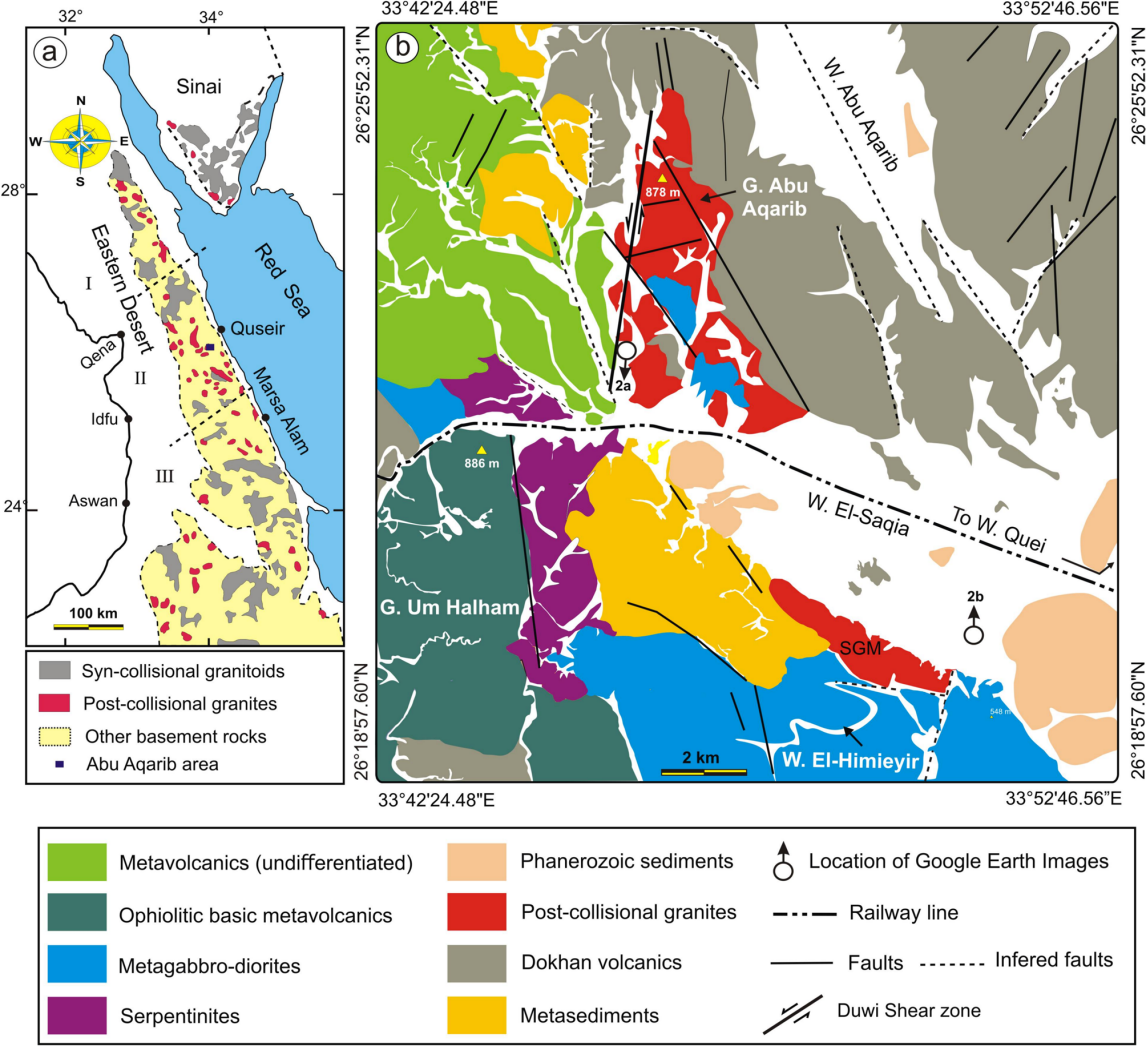
(**a**) Simplified regional geological map showing the distribution of Egyptian granitoid rocks (older and younger) in the Eastern Desert and Sinai. The map was prepared using CorelDRAW Graphics Suite 2018 with Google Earth Pro desktop (version 7.3.6; https://earth.google.com/) as the base image, based on the geological information of Greenberg^81^ for the Eastern Desert and El-Shazly et al.^82^ for Sinai. Fields I, II, and II represent the northern, central, and southern Eastern Desert terranes, respectively. (**b**) Geologic map of the Abu Aqarib area. The map was prepared using CorelDRAW Graphics Suite 2018 with Google Earth Pro desktop (version 7.3.6; https://earth.google.com/) as the base image. Geological boundaries and rock-unit distribution were compiled from EGSMA^83^. SGM: southern granitic mass.

**Fig. 2:**
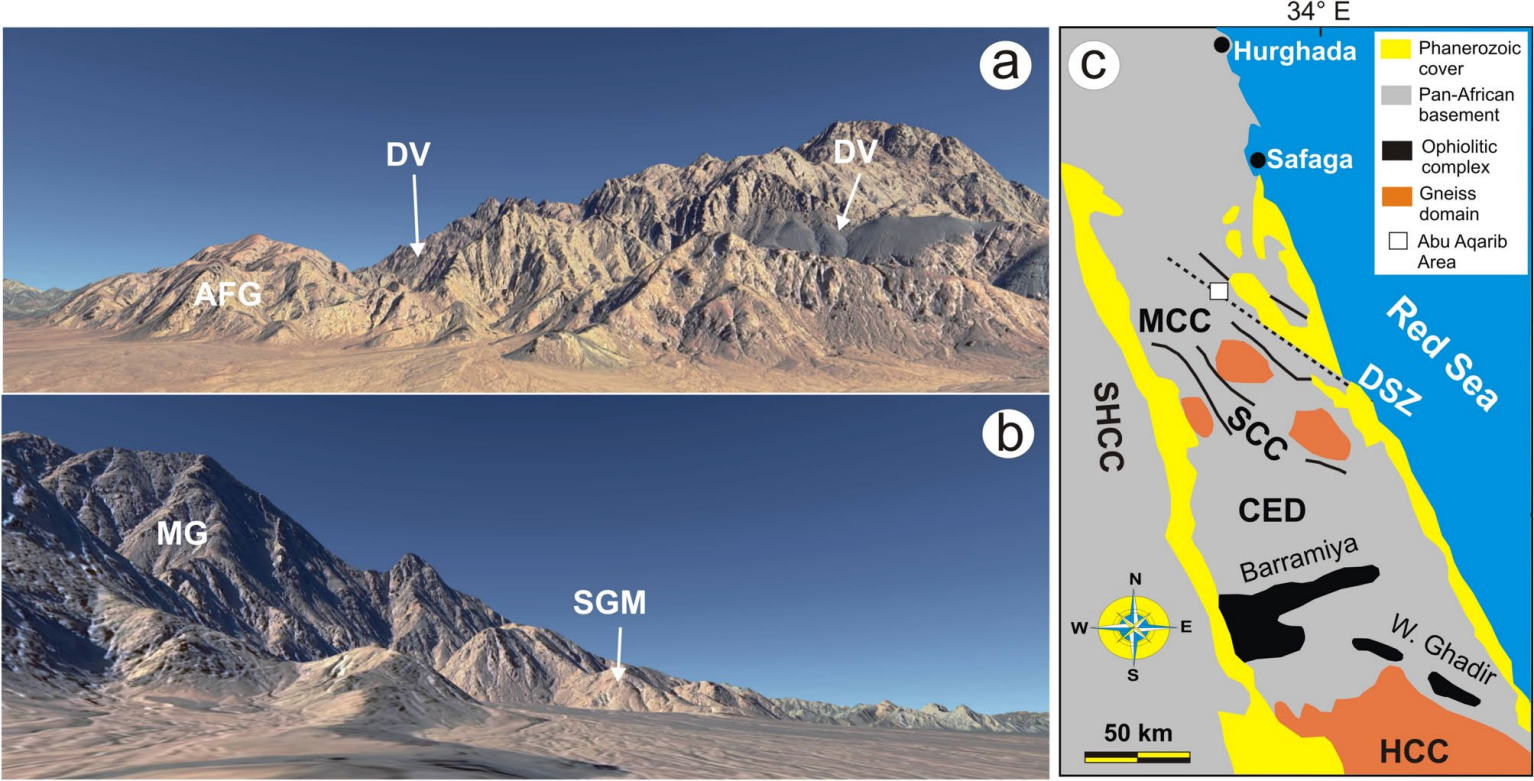
(**a**,**b**) Google Earth Pro desktop images (version 7.3.6; https://earth.google.com/) illustrating the field relationships of the Abu Aqarib A-type granites (AFGs). **a** Abu Aqarib AFGs intruding Dokhan volcanics (DV). (**b**) Southern granitic mass (SGM) enclosed within rounded metagabbro (MG). (**c**) Simplified tectonic map of the Pan-African basement of southeastern Egypt and northeastern Sudan. The map was prepared using CorelDRAW Graphics Suite 2018 with Google Earth Pro desktop (version 7.3.6; https://earth.google.com/) as the base image, based on the regional geological framework of Abd El-Wahed^84^. NED: Northern Eastern Desert, CED: Central Eastern Desert, MCC: Meatiq core complex, SCC: Sibai core complex, HCC: Hafafit core complex, and SHCC: El-Shalul core complex.

The Abu Aqarib area is located in the CED, about 45 km southwest of Safaga City (Fig. 1a). The area is mainly covered by basement rocks, overlain along southeastern sides by Phanerozoic sediments. The basement rocks, from younger to older, comprise alkali-feldspar granites, Dokhan volcanics, Hammamat sediments, metasediments, metavolcanics (undifferentiated), ophiolitic basic metavolcanics, metagabbros, and serpentinites (Fig. 1b). The granitic rocks form two principal masses separated by Wadi El-Saqia: a northern, elongate oval-shaped body (~ 7 km^2^; Gabal Abu Aqarib) and a smaller southern body (~ 3 km^2^). Both masses form rugged mountainous terrain, reaching up to 878 m above sea level. The northern pluton intrudes Dokhan volcanics (Fig. 2a), whereas the southern pluton cuts across metasediments, metavolcanics, metagabbro, and Dokhan volcanics (Fig. 2b), both with sharp intrusive contact. The two masses are very similar, having a pink color with a reddish tint, and are commonly cut by NE-trending quartz veins of high radiometric anomalies^15^, acidic dikes, and small pegmatitic pockets. Weathering produces boulder-type disintegration in granitic rocks, particularly along the western periphery.

The area is strongly deformed by two main fault systems^14^. A major WNW–ESE fault (the Saqia–Missikat Fault; SMF) extends from the Red Sea westward through Wadi Saqia into the Phanerozoic cover. South of this fault, the terrain is dominated by NW–SE-trending faults and folds. In addition, the granitic pluton is traversed by NW faults of the Najd Fault System and lies within the Duwi shear zone, where shearing and alteration features are locally observed (Fig. 2c). These fractures act as feeding channels for the hydrothermal solutions, which infiltrate through granitic rocks and cause metasomatic alteration.

## Petrography

Microscopic investigation reveals that the studied rocks record two genetically related paragenetic stages: (1) hypersolvus alkali feldspar granite (AFG) representing the primary magmatic assemblage and (2) carbonate episyenite reflecting intense late- to post-magmatic hydrothermal alteration.

### Hypersolvus AFG

The hypersolvus AFG is predominantly composed of K-feldspar (53.70‒70.20%) and quartz (32.20‒46.70%), with subordinate plagioclase and minor mafic minerals represented by biotite and amphibole. Accessory minerals comprise zircon, epidote, rutile, and Fe–Ti oxides, while secondary phases include sericite, chlorite, and titanite.

**Fig. 3:**
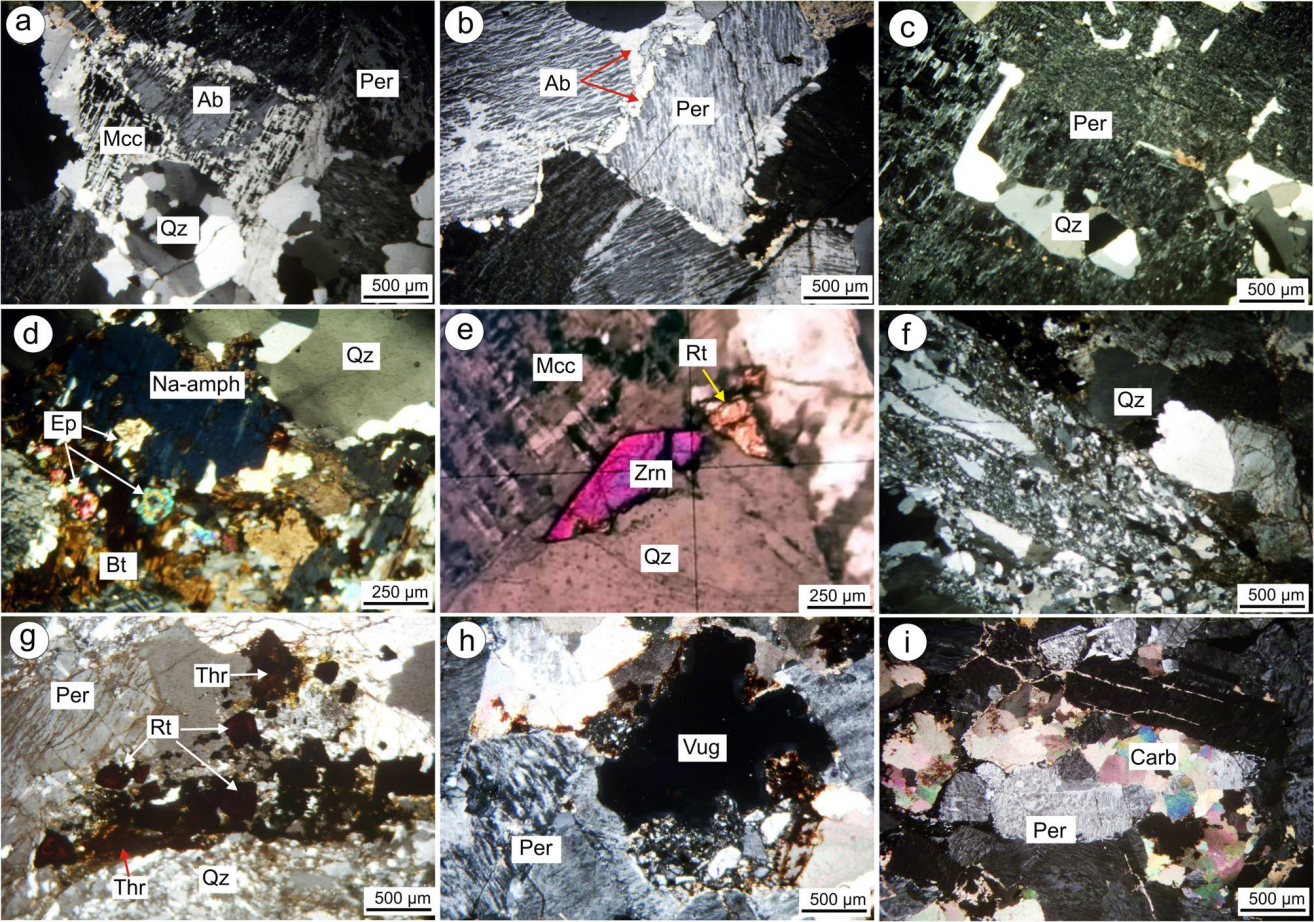
Photomicrographs of Abu Aqarib AFGs and associated episyenite in C.N. (**a**) Microcline (Mcc) showing exsolved lamella of albite (Ab). (**b**) Perthite (Per) rimmed by thin albite (ab) bands. (**c**) Graphic intergrowth between quartz and K-feldspar. (**d**) Prismatic sodic amphibole associated with biotite and epidote aggregates. (**e**) Prismatic zircon crystal and rutile included within K-feldspar. (**f**) Carbonate episyenite showing intense deformation and mylonitic texture. (**g**) Clusters of rutile and thorite filling open spaces in carbonate episyenite as a result of deformation. (**h**) Strongly altered carbonate episyenite showing vuggy porosity due to quartz dissolution (desilicification). (**i**) Carbonate infilling of cavities and fractures produces amygdaloidal texture.

K-feldspar occurs as subhedral to euhedral tabular crystals, mainly represented by orthoclase with subordinate microcline. It commonly exhibits well-developed perthitic textures, characterized by exsolved albite lamellae and blebs arranged in characteristic fish-bone patterns (Fig. 3a). The perthitic K-feldspar is commonly bordered by thin albite rims (Fig. 3b), reflecting late-stage subsolidus re-equilibration during cooling. Quartz occurs as subhedral to anhedral interstitial grains filling the spaces between K-feldspar crystals. It commonly exhibits undulose extinction, indicating post-crystallization deformation. Locally, graphic intergrowths between quartz and K-feldspar are observed (Fig. 3c), suggesting simultaneous crystallization from a silica-saturated melt during the late magmatic stage.

Plagioclase occurs as subhedral to anhedral prismatic crystals, commonly exhibiting corrosion textures due to interaction with both quartz and K-feldspar. Amphibole is a minor phase, occurring as prismatic crystals with a characteristic blue color (Na-amphibole), and is closely associated with biotite and epidote (Fig. 3d). Biotite often occurs in association with iron oxides and is partially to completely altered to chlorite. Zircon and rutile occur as inclusions within quartz and K-feldspar (Fig. 3e).

### Carbonate episyenite

Carbonate episyenite represents a highly altered equivalent of the hypersolvus AFG, formed through intense hydrothermal metasomatism coupled with brittle-ductile deformation. Petrographic observations reveal extensive fracturing, fissuring, and cataclastic to mylonitic textures (Fig. 3f), as well as the development of vugs and cavities. These features are diagnostic of episyenitization and record a complex sequence of fluid–rock interaction processes. The rock is dominated by K-feldspar and albite, with minor relict quartz. Secondary minerals include carbonate, chlorite, and illite, whereas accessory phases are zircon, rutile, and thorite (Fig. 3g).

The earliest stage of alteration is characterized by pervasive quartz dissolution (desilicification), driven by the infiltration of alkaline hydrothermal fluids enriched in Na and K. This process results in the formation of a highly porous rock marked by abundant vugs and cavities (Fig. 3h), representing a key step in episyenite formation and significantly modifying the primary granite mineralogy. Albitization (Na-metasomatism) constitutes a major alteration process, leading to the extensive replacement of K-feldspar and partial illitization of earlier-formed K-feldspars. Concurrently, ferromagnesian minerals, particularly amphiboles, are completely dissolved and replaced by secondary phases, reflecting the aggressive nature of the circulating alkaline fluids. The final stage of alteration is characterized by the precipitation of carbonate minerals within voids and fractures, producing characteristic amygdaloidal textures (Fig. 3i).

## Methodology

Microprobe analyses of selected mineral phases from the Abu Aqarib granites (Suppl. Table 1) were carried out using a CAMECA SX100 electron probe at the Department of Geosciences, University of Oslo. Analytical conditions were 15 kV accelerating voltage, 15 nA beam current, a 2 μm beam diameter, 10 s of peak count duration, and 5 s for both low and high backgrounds. Data correction was performed using ZAF matrix correction procedures in conjunction with the CITZAF protocol. Calibration was achieved using a mixture of natural and synthetic mineral standards to ensure analytical accuracy.

A total of 11 representative samples of Abu Aqarib granites were selected for whole-rock geochemical analysis (Suppl. Table 2). Samples were crushed into homogenized pebble-sized particles and subsequently pulverized using an agate mill to obtain homogeneous powders. Major elements and selected trace elements were determined by X-ray fluorescence (XRF) using a ThermoARL XRF spectrometer. For analysis, powdered samples were mixed with di-lithium tetraborate flux in a 1:2 ratio and fused at 1000 °C to produce homogeneous glass beads. These beads were reground, re-fused, and polished on a diamond lap to obtain flat, smooth surfaces suitable for precise measurement. Calibration was performed using the USGS reference material GSP-2 (650CC). Loss on ignition (LOI) was determined gravimetrically based on weight loss after heating at 1000 °C.

Rare earth elements (REEs) and additional trace elements were analyzed using inductively coupled plasma mass spectrometry (ICP-MS; Agilent 7700). Approximately 50 mg of each powdered sample was digested in acid-washed Teflon vessels by refluxing in a 3:1 mixture of nitric and hydrofluoric acids at 250 °C for a minimum of 8 h. Instrument calibration curves were established using blank fused beads prepared from the same flux batch as the unknown samples, alongside USGS reference materials AGV-2 and RGM-2. To control quality, additional USGS standards (DTS-2, BCR-1, and G-2) were included as unknown to monitor analytical accuracy and precision. Both XRF and ICP analyses were performed at the GeoAnalytical Lab, Washington State University (WSU), Pullman, WA, USA. Analytical precision, accuracy and detection limits follow those reported by Johnson et al.^16^.

Petrogenetic interpretations are based exclusively on the least altered hypersolvus AFG samples, identified based on petrographic observations. The altered carbonate episyenite sample was excluded from tectonic discrimination, magma source, and temperature calculations because its major- and trace-element composition may have been modified during episyenitization. This sample was used only to evaluate fluid-rock interaction and element mobility through isocon analysis.

Petrographic examination of all available carbonate episyenite samples (n = 5) revealed consistent mineralogical and textural characteristics. Therefore, for whole-rock geochemical analysis, a single representative sample was selected from this suite. However, additional whole-rock geochemical analyses would be valuable to further evaluate the magnitude and variability of trace-element redistribution during episyenitization.

## Mineral chemistry

Representative chemical compositions and structural formulas of feldspar (plagioclase and K-feldspar), chlorite, carbonate, Fe-Ti oxides, zircon, and thorite are presented in Suppl. Table 1a-g. K-Feldspar, plagioclase, zircon, and iron oxides are analyzed in both hypersolvus AFG and carbonate episyenite, whereas chlorite, carbonate, and thorite pertain only to carbonate episyenite.

**Fig. 4:**
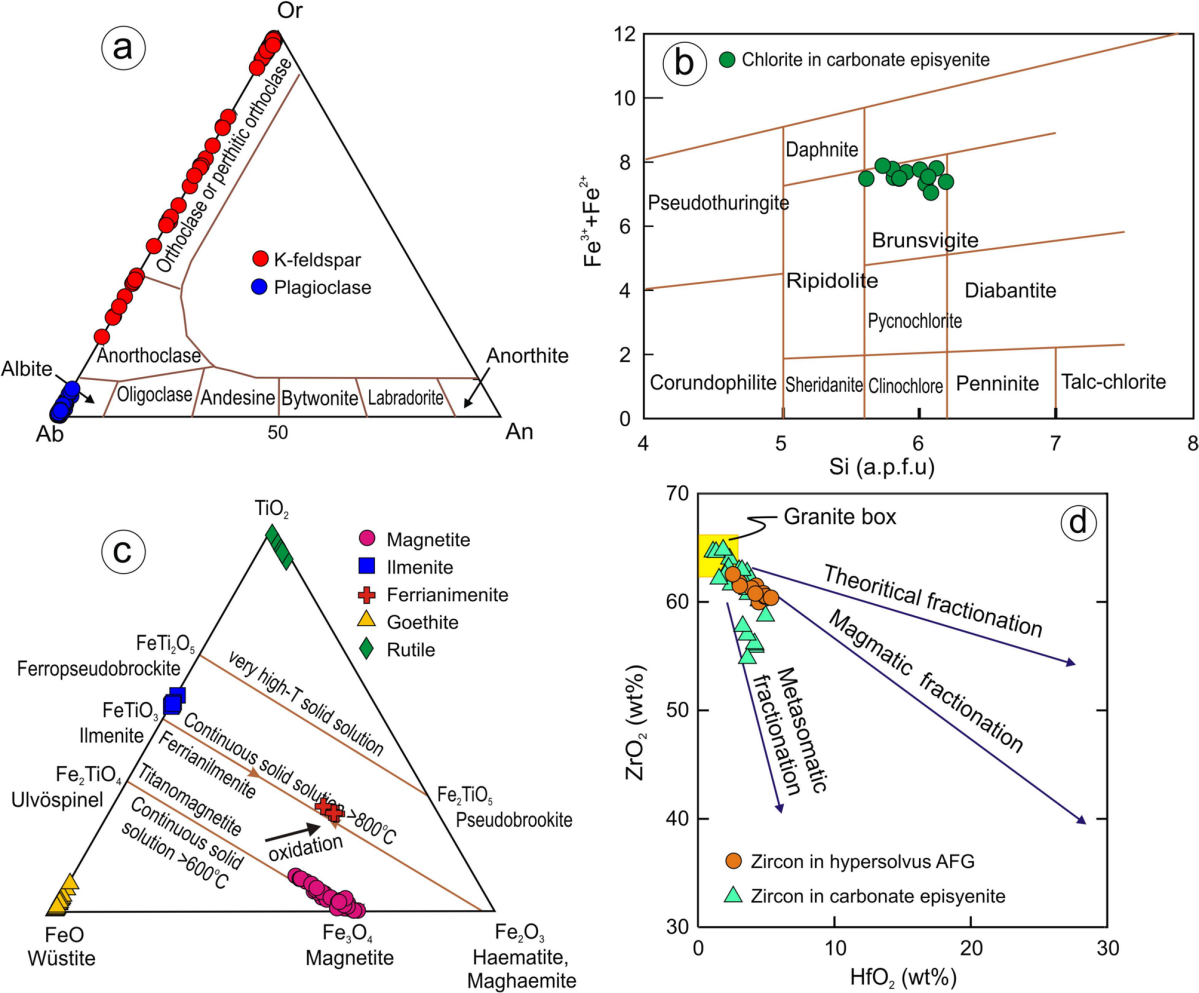
Mineral chemistry diagrams. (**a**) Or-Ab-An classification diagram for feldspars from Abu Aqarib AFGs^85^. (**b**) Si–(Fe^2+^ + Fe^3+^) classification diagram for chlorite^18^ in carbonate episyenite. (**c**) TiO_2_-FeO-Fe_2_O_3_ ternary diagram showing compositions and equilibrium tie lines between analyzed ilmenite and magnetite ^19^. (**d**) ZrO_2_ vs. HfO_2_ diagram for zircons from Abu Aqarib AFGs and carbonate episyenite. Zircon fractionation trends are from Kempe^86^.

*K-feldspar* in both the hypersolvus AFG and carbonate episyenite (Suppl. Table 1a) exhibits a wide range of Na_2_O (~ 0.3‒8.6 wt% and 0.2‒9.1 wt%, respectively) and K_2_O (~ 4.6‒16.2 wt% and 3.6‒16.7 wt%, respectively) with consistently low CaO content (> 0.2 wt%). It ranges in composition from anorthoclase (Or_21-36.5_) to perthitic orthoclase (Or_44-98_) (Fig. 4a). The elevated Na₂O contents and variable Na₂O/K₂O ratios (0.02‒1.87 in hypersolvus AFG and 0.01‒2.53 in carbonate episyenite) may indicate alkali exchange. These compositions likely reflect a co-existence of primary alkali-feldspar crystallization and subsequent Na metasomatism, which appears to be more pronounced in the carbonate episyenite.

*Plagioclase* (Suppl. Table 1b) is limited to the albite end member (Fig. 4a) ranging from Ab_94–99_ in the hypersolvus AFG and Ab_93–99_ in the carbonate episyenite. It is characterized by high Na_2_O (~ 11–12 wt% in hypersolvus AFG and 11–13 wt% in carbonate episyenite) and very low CaO (< 0.25 wt%) contents. The low CaO/Na₂O ratio (up to 0.02 in both hypersolvus AFG and carbonate episyenite) further supports secondary replacement rather than primary magmatic crystallization^17^.

*Chlorite* in carbonate episyenite (Suppl. Table 1c) is Fe-rich with high FeO contents (~ 36–40 wt%) and low Mg# (0.1–0.2). Based on the classification scheme of Hey^18^, chlorite is compositionally classified as brunsvigite (Fig. 4b). Chlorite thermometry yields temperatures of 231‒325 °C (Suppl. Table 1c), consistent with alteration under low- to moderate-temperature hydrothermal conditions.

*Carbonate* minerals are represented by calcite and dolomite and occur exclusively in the carbonate episyenite (Suppl. Table 1d). Calcite is dominated by CaCO_3_ (~ 93–99%), with minor MgCO_3_ (~ 0.6–7%) and FeCO_3_ (up to 1.35%). In contrast, dolomite exhibits a wide compositional range, with MgCO_3_ ≈ 25–46% and CaCO_3_ ≈ 49–71%.

*Fe-Ti oxides* comprise magnetite, ilmenite, goethite, and rutile (Suppl. Table 1e). Ilmenite composition ranges from stoichiometric ilmenite to ferrianilmenite (Fig. 4c). The latter exhibits lower TiO_2_ (~ 22‒25 wt%) and higher FeO (~ 61‒66 wt%) contents compared to stoichiometric ilmenite (TiO_2_ ≈ 52‒55 wt% and FeO ≈ 42‒45 wt%). Likewise, magnetite varies from stoichiometric magnetite to titanomagnetite containing ~ 2‒9 wt% TiO₂, indicating partial solid solution toward ulvöspinel^19,20^. Goethite, which is associated with alteration, exhibits low values (~ 76‒89 wt %) and a wide range of FeO* (~ 68‒81 wt%) and SiO_2_ (2.8‒8 wt%). Rutile contains 88‒92 wt% TiO_2_ with minor FeO (1.4‒7.5 wt%).

*Zircon* from the hypersolvus AFG (Suppl. Table 1f) contains ~ 60–63 wt% ZrO₂, ~ 31–33 wt% SiO₂, and ~ 2.6–5.4 wt% HfO₂, consistent with typical magmatic zircon compositions^21^. Zircon from the carbonate episyenite shows broadly similar compositions (ZrO₂ ≈ 55–65 wt%, SiO₂ ≈ 29–36 wt%, and HfO₂ ≈ 1–5 wt%), suggesting that it largely preserves its original magmatic signature despite subsequent hydrothermal alteration. On the ZrO₂–HfO₂ diagram (Fig. 4d), zircon analyses from both hypersolvus AFG and carbonate episyenite plot within or close to the granite field, indicating only limited post-magmatic modification.

*Thorite* (Suppl. Table 1g), which occurs only in carbonate episyenite, shows a relatively uniform composition, with ThO₂ contents of ~ 56‒63 wt% and SiO₂ contents of ~ 14‒16 wt%. It contains notable concentrations of heavy rare earth elements (HREEs), including Yb_2_O_3_ (up to 7 wt%), Er_2_O_3_ (up to 3 wt%), and Dy_2_O_3_ (up to 4 wt%), as well as elevated F concentration (up to 4430 ppm). Significant P₂O₅ (~ 5 wt%) and CaO (~ 2 wt%) contents indicate coupled substitution processes and partial alteration toward thorogummite. The metamict nature of thorite likely enhanced its susceptibility to fluid-mediated modification, facilitating the incorporation of additional elements during post-magmatic alteration. Consequently, its present composition reflects both primary crystallization and subsequent hydrothermal overprinting.

## Whole-rock geochemistry

### Geochemical characteristics

Whole-rock geochemical data (major, trace elements, and REEs) and CIPW normative compositions of the Abu Aqarib hypersolvus AFGs and associated carbonate episyenite are presented in Supplementary Table 2.

The Abu Aqarib hypersolvus AFGs are characterized by high SiO_2_ contents (~ 70.7–76.5 wt%), elevated total alkalis (Na_2_O + K_2_O = 6.89–8.60 wt%), and an extremely high FeO^T^/(FeO^T^ + MgO) ratio (0.94‒0.99). In contrast, they are markedly depleted in CaO (0.01–1.55 wt%) and MgO (0.01–0.13 wt%), with correspondingly low Mg# values (0.01–0.10). The carbonate episyenite displays a distinct geochemical signature, characterized by lower SiO₂ (48.43 wt%) and higher CaO (6.54 wt%) and MgO (3.91 wt%) contents, along with a significantly lower FeO^T^/(FeO^T^ + MgO) ratio (0.48). These compositional variations are consistent with intense hydrothermal modification, involving silica leaching and carbonate addition.

**Fig. 5:**
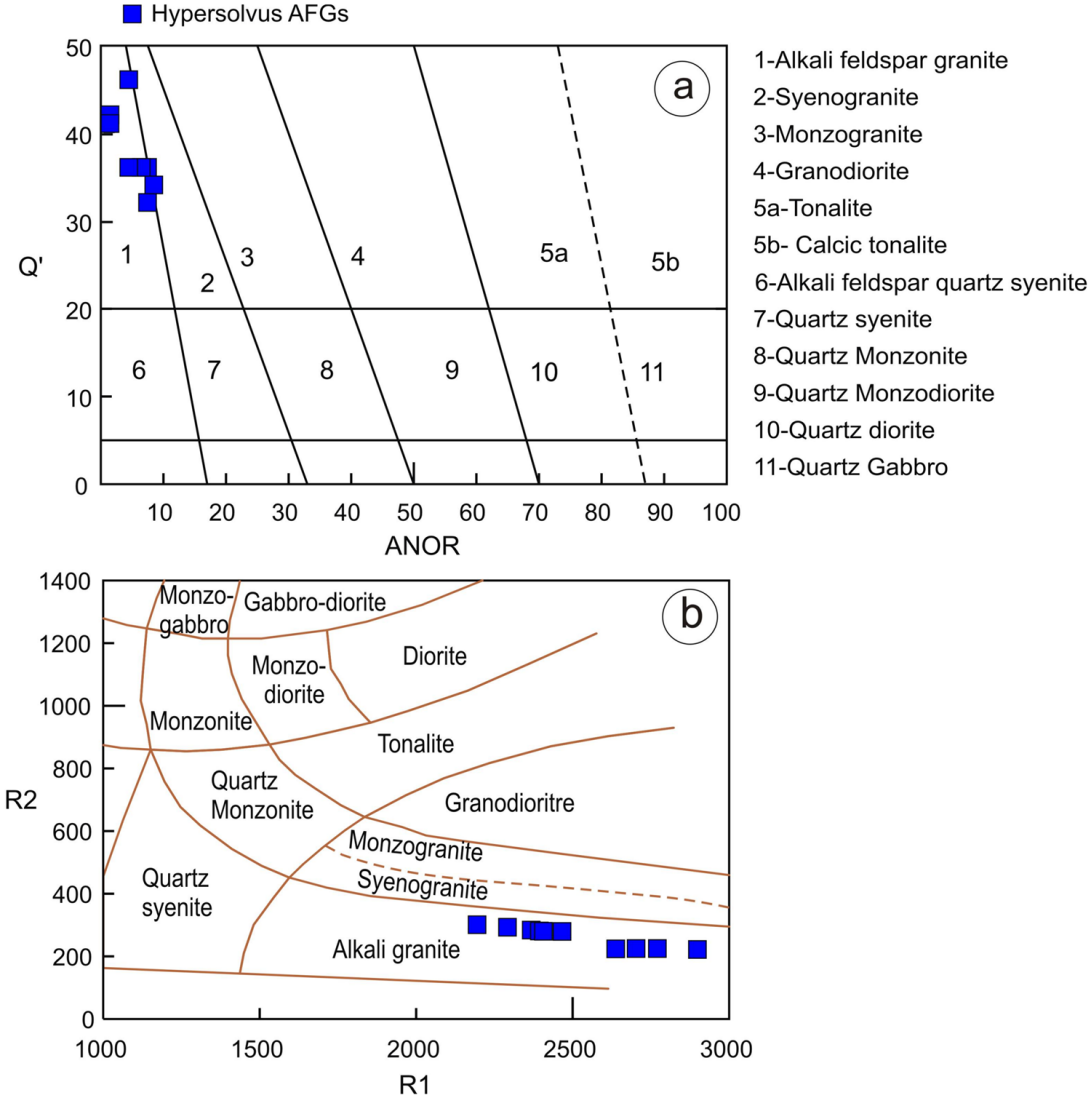
Major-element classification diagrams for Abu Aqarib granites. (**a**) The Q′-ANOR classification diagram for plutonic rocks^87^; (**b**) R1-R2 discrimination diagram of plutonic rocks^88^.

On the Q-ANOR normative classification diagram (Fig. 5a), the hypersolvus AFG samples plot in the alkali feldspar granite field. This classification is further confirmed by the multicationic R1-R2 discrimination diagram (Fig. 5b), where all samples occupy the same compositional field.

**Fig. 6:**
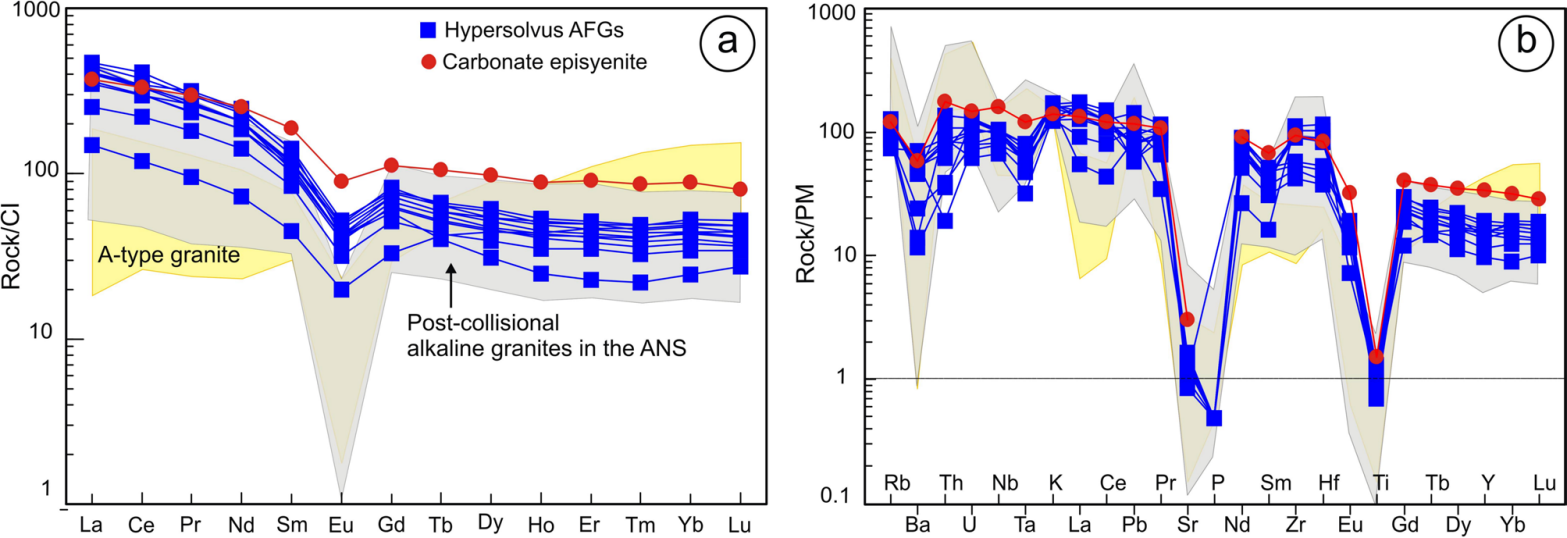
Trace and REE compositions of Abu Aqarib granites. (**a**) Chondrite (CI)-normalized REE patterns. (**b**) Primitive mantle (PM) normalized trace-element patterns. CI and PM normalized values are from McDonough and Sun^22^. The fields of A-type granite in ED, Egypt^69,89,90^ and post-collisional alkaline/peralkaline granites in the ANS^23,25,26,28,29,91^ are plotted for comparison.

The hypersolvus AFGs are enriched in incompatible elements, particularly F (2293–4430 ppm), Zr (~ 444–1180 ppm), and total REEs (~ 197–579 ppm). Their REE abundances are 79–232 times chondrite values (ΣREE = 2.5^22^,). Their chondrite (CI)-normalized REE patterns (Fig. 6a) show fractionation trends ((La/Lu)_N_ = 3.11–10.24), with significant enrichment in the light REEs (∑LREEs ≈ 164‒540 ppm; (La/Yb)_N_ = 3.06‒9.6) relative to heavy REEs (∑HREEs ≈ 26‒65 ppm; (Gd/Yb)_N_ = 0.68‒1.74). All samples display slight to moderate negative Eu anomalies (Eu/Eu* = 0.42–0.52), consistent with crystallization from a primitive melt already depleted in plagioclase, or alternatively, with magma generation through partial melting of a differentiated source^23^. The primitive mantle (PM)-normalized multi-element spider diagram (Fig. 6b) reveals enrichment in most incompatible elements relative to PM with pronounced negative anomalies in Ti, Sr, and P, typical of A-type granite signature^24^. The trace element and REE signatures of Abu Aqarib hypersolvus AFGs closely resemble those of alkaline granites in the ANS^7,23,25–29^ (Fig. 6). The carbonate episyenite exhibits CI- and PM-normalized patterns that broadly overlap with those of the hypersolvus AFGs (Fig. 6a, b).

### Magma type and tectonic setting

Multiple lines of evidence indicate that the Abu Aqarib hypersolvus AFGs largely retain their primary magmatic signatures. These include (1) low loss on ignition (LOI = 0.58–1.52 wt%) and lack of Ce anomalies (Fig. 6a), indicating minimal post-magmatic alteration; (2) chemical index of alteration values (CIA ≈ 49‒51; Suppl. Table 2) that fall within the range reported for fresh, unaltered granites (45–55;^30^); (3) Normative mineral compositions (Ab ≈ 29–36, Q ≈ 31–43, Or ≈ 22–31, Suppl. Table 2) which further supports limited secondary modification^31^, and the preservation of primary magmatic textures, including perthitic and micrographic intergrowths (Fig. 3).

**Fig. 7:**
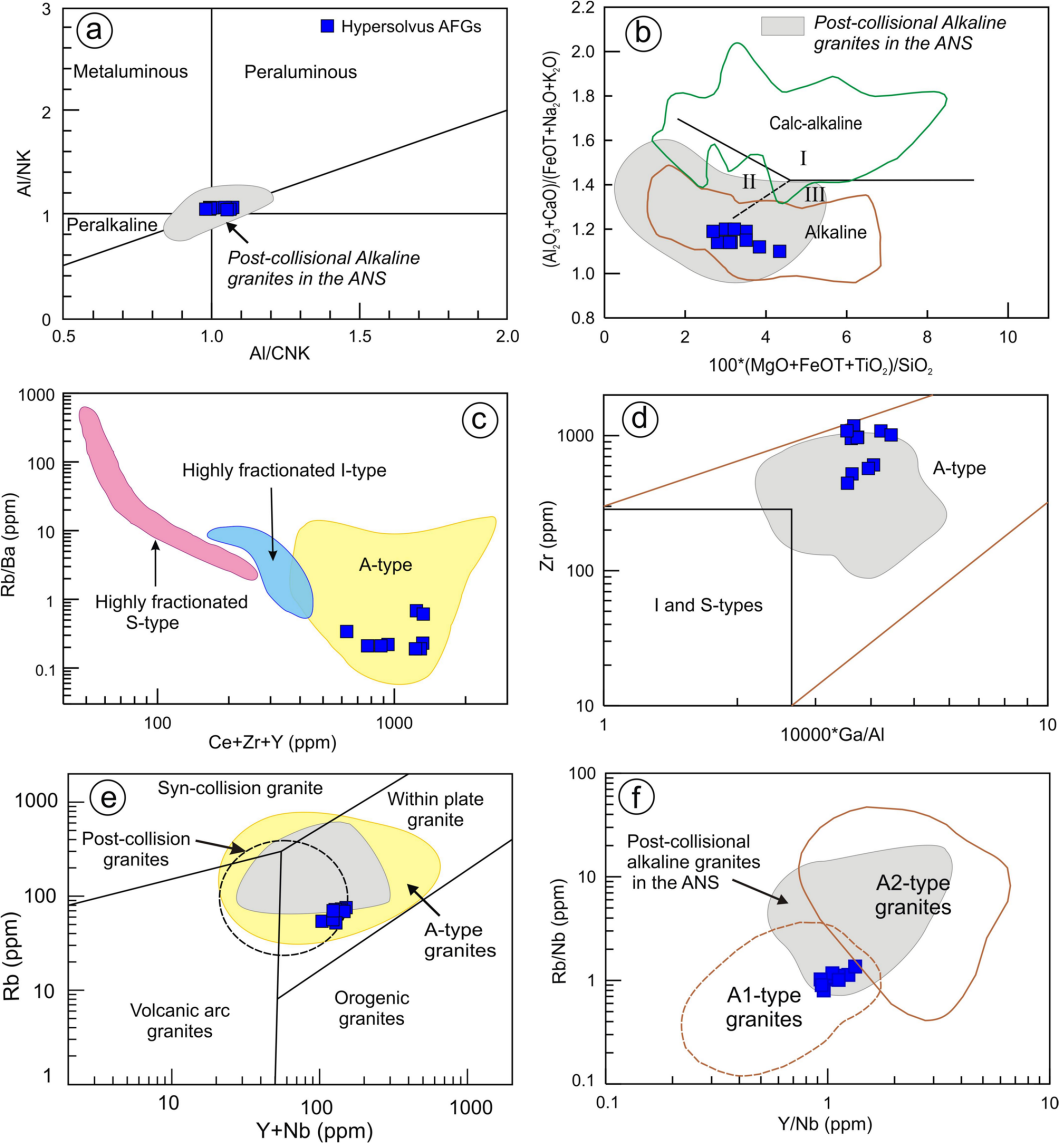
Magma type and tectonic setting discrimination diagrams for Abu Aqarib granites. **a** A/CNK versus A/NK binary diagram^92^. A/CNK = molar Al_2_O_3_/(Na_2_O + K_2_O) and A/NK = Al_2_O_3_/(CaO + Na_2_O + K_2_O). **b** Discrimination diagram of Sylvester^93^ for distinguishing between calc-alkaline and strongly peraluminous granites (I), highly fractionated calc-alkaline granite (II), and alkaline granites (III). The calc-alkaline and alkaline fields of the ANS are adapted from Be’eri-Shlevin et al.^94^. **c**, **d** Ce + Zr + Y vs. Rb/Ba and Zr vs. 1000* (Ga/Al) diagrams, for A-type granites, respectively^35^. **e** Rb vs. Y + Nb tectonic discrimination diagram^39^. **f** Rb/Nb vs. Y/Nb diagram^38^ to distinguish between A1 and A2 granites. Fields of post-collisional granite^40^, A-type granite^35^, and post-collisional alkaline granites in the ANS ^23,25,26,28,29,91^ are plotted for comparison.

The hypersolvus AFGs show high SiO_2_ and K_2_O contents and elevated FeO^T^/(FeO^T^ + MgO) ratios, characteristic of ferroan granites^32^. They exhibit metaluminous to slightly peraluminous affinity (Fig. 7a) with an alumina saturation index (ASI) = 0.98‒1.06. Their agpaitic index ranges from 0.93 to 0.95, typical of alkaline granite^33^. This is consistent with the discrimination diagram of Sylvester (1989), where samples plot in the field of alkaline granites (Fig. 7b). Abu Aqarib granites also exhibit Rb/Sr ratios greater than unity (Suppl. Table 2), comparable to other alkaline granites in the ANS^25,26,34^. Furthermore, feldspar chemistry (Suppl. Table 1a, b) indicates the dominance of K–Na feldspars, including orthoclase, anorthoclase, and albite. Their low CaO/(Na₂O + K₂O) ratios (~ 0.002–0.006), compared to those of the host rocks (~ 0.04, Suppl. Table 2), further support their alkaline nature^23^.

Hypersolvus AFGs exhibit significant enrichment in incompatible elements such as Zr (~ 831 ppm), Nb (~ 63 ppm), Y (~ 67 ppm), Ce (~ 187), F (3365 ppm), and ∑REE (~ 484 ppm), consistent with A-type granites^23,24,35–37^. This A-type affinity is further confirmed on several discrimination diagrams, including the Y + Ce + Zr (ppm) versus Rb/Ba diagram (Fig. 7c) and the Zr vs. (100,000 * Ga/Al) diagram (Fig. 7d). The absence of pronounced negative Nb anomalies in PM-normalized spider diagrams (Fig. 6b) and low La/Nb ratios (< 2; Suppl. Table 2) are more consistent with intraplate alkaline magmatism^38^ and argue against a subduction-related origin. Subduction-related magmas commonly exhibit marked Nb depletion and elevated La/Nb ratios because slab-derived fluids preferentially enrich large-ion lithophile elements (LILEs), whereas Nb remains relatively immobile in residual phases^39,40^.

The REE patterns of hypersolvus AFGs are strongly fractionated (Fig. 6a), with LREE enrichment and moderate negative Eu anomalies (Eu/Eu* = 0.42–0.52), suggesting significant feldspar fractionation during magma evolution. Low concentrations of Cr, Sc, V, Ba, and Sr (Suppl. Table 2) also indicate removal of feldspar and mafic minerals during fractional crystallization.

The tectonic setting of A-type granites in the ED, including Abu Aqarib AFGs, remains debated, particularly regarding their formation in post-collisional versus anorogenic extensional regimes^33,35,36^. On the Y + Nb versus Rb diagram, Abu Aqarib AFGs plot within the post-collisional A-type granite field (Fig. 7e). However, the classification scheme of Eby^38^ reveals a more complex signature. This scheme distinguishes between A1-type granites, related to anorogenic within-plate settings, and A2-type granites, associated with post-collisional environments. Most samples yield Y/Nb ratios < 1.2 (Suppl. Table 2) and fall predominantly within the A1 field on the Rb/Nb versus Y/Nb diagram (Fig. 7f), although they show a clear tendency toward the A2 domain. Similarly, their positions along the boundary between A1 and A2 fields on the Nb–Y–Zr/4 ternary diagram (Suppl. Figure 1a) suggest a hybrid geochemical affinity.

**Fig. 8:**
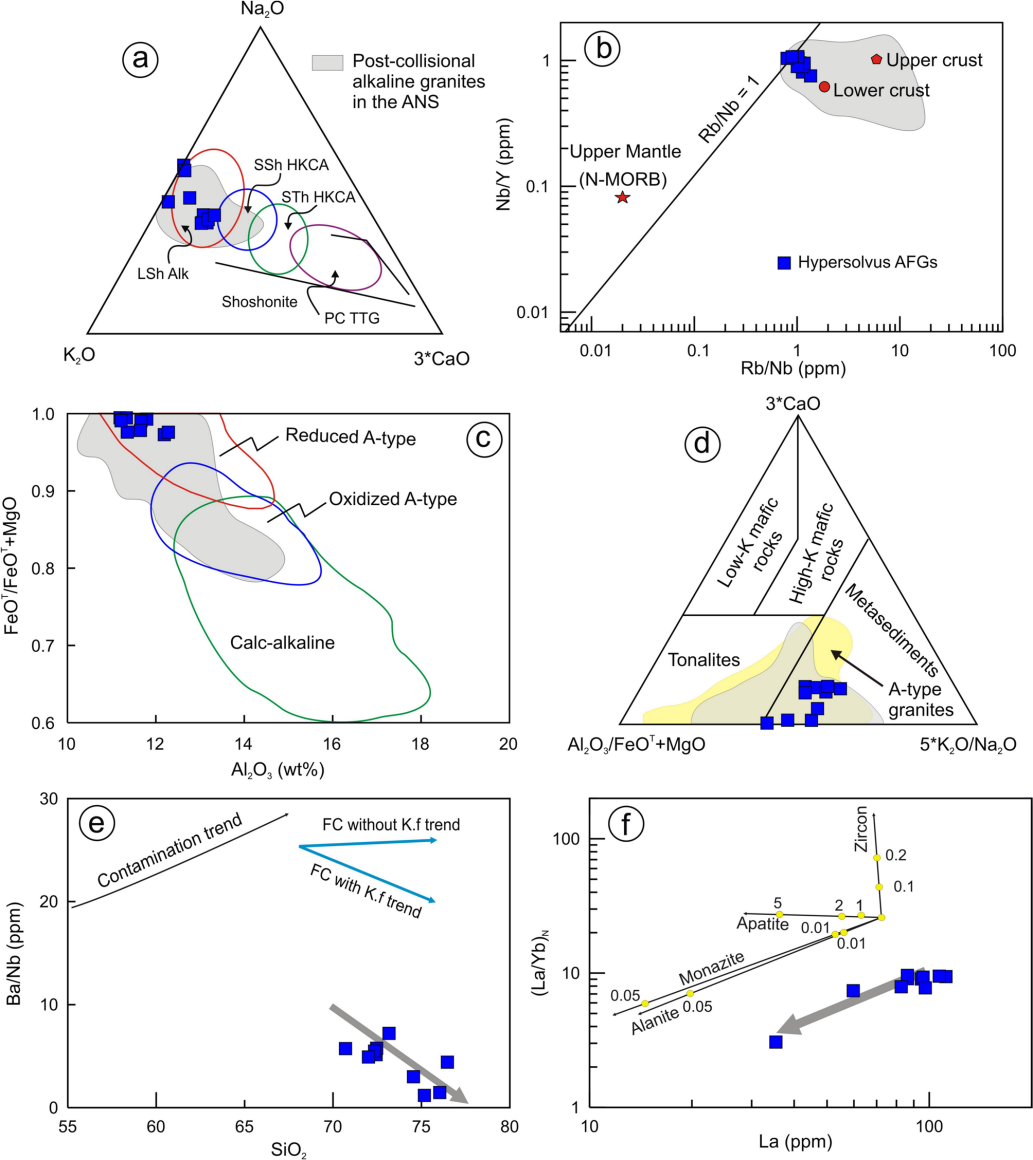
Petrogenesis discrimination diagrams for Abu Aqarib granites. (**a**) K_2_O‒ Na_2_O‒ 3 × CaO ternary diagram^95^. LS Alk: late-shear alkaline granite series; SSh: syn-shear granite series; STh: syn-thrust granite series; TTG: trondhjemite tonalite; and PC: pre-collision. (**b**) Rb/Y vs. Nb/Y diagram^96^. The compositions of the lower and upper crust are adapted from Rudnick and Gao^56^, while the upper mantle (N-MORB) value is from Sun and McDonough^97^. (**c**) FeO^T^/(FeO^T^ + MgO) vs. Al_2_O_3_ diagram. Reduced, oxidized A-type magma, and calc-alkaline fields are from Dall’Agnol and de Oliveira^98^. (**d**) Al_2_O_3_/(FeO^T^ + MgO) ‒ 3 × CaO ‒ 5 × (K_2_O/Na_2_O) source discrimination diagram^99^. (**e**) Ba/Nb vs. SiO_2_ diagram. Fractionation trends with K-feldspar and without K-feldspar, and contamination trends are from Hubbard et al.^100^ (**f**) (La/Yb)_N_ vs. La diagram showing the change of REE patterns by separation of accessory minerals, especially allanite and apatite. Chondrite-normalized values are from Sun and McDonough^97^, partition coefficients are from Fujimaki^101^ for apatite, Mahood and Hildreth^102^ for zircon and allanite, and Yurimoto et al.^103^ for monazite. Fields of A-type granite^35^, and post-collisional alkaline granites in the ANS^23,25,26,28,29,91^ are plotted for comparison.

On the K_2_O‒ Na_2_O‒ 3 × CaO diagram (Fig. 8a), the samples occupy the field of the late-shear alkaline granite series. Such a classification suggests emplacement during a late-to post-tectonic stage associated with major shear zone development. These deep-seated shear zones facilitated magma ascent and acted as conduits for evolved melts derived from the lower crust^41^.

Overall, the geochemical characteristics of the Abu Aqarib AFGs are consistent with emplacement in a post-collisional extensional setting. Their transitional A1–A2 affinity indicates formation during the transition from compressional tectonics to post-collisional extension, similar to many Neoproterozoic A-type granites in the ANS. However, in the absence of direct zircon U–Pb age data, any correlation with the late Neoproterozoic post-collisional magmatic episode (ca. 590–540 Ma) remains tentative and is based on regional geological relationships rather than direct geochronological evidence.

### Crystallization conditions: temperature, pressure, and redox state

The Abu Aqarib hypersolvus AFGs crystallized under high-temperature, shallow crustal, and reduced conditions, consistent with A-type granite. Zircon saturation thermometry^42^ yields elevated temperatures (Tzr ≈ 875‒1018 °C) with an average of 938 °C (Suppl. Table 2), indicative of hot, dry, and evolved silicic melts^43,44^. Comparable temperatures (Tzr ≈ 900‒929 °C) have been reported for post-collisional A-type granites elsewhere in the ANS^24,25^. In fluorine-rich systems, zircon saturation temperatures may be slightly overestimated due to increased zircon solubility^42,44^. In addition, zircon saturation thermometry assumes that whole-rock Zr concentrations primarily reflect magmatic processes. However, inherited zircon populations may locally increase the bulk-rock Zr budget and potentially result in overestimated temperatures^45^. As zircon imaging and U–Pb geochronological analyses were not performed in this study, the presence and significance of inherited zircon cores cannot be directly evaluated. Furthermore, whole-rock Zr concentrations may be modified during magmatic differentiation, as progressive zircon crystallization during fractional crystallization can deplete residual melts in Zr and influence calculated saturation temperatures^42^. Consequently, the calculated Tzr values are regarded as approximate estimates of magma thermal conditions and likely reflect initial melt temperatures rather than true liquidus conditions.

In contrast, mineral-based thermometry records significantly lower temperatures, reflecting subsolidus re-equilibration during progressive cooling. Coexisting K-feldspar–albite pairs yield temperatures between 451 and 740 °C^46^, while magnetite–ilmenite pairs indicate an average temperature of about 503 °C^47^.

Pressure estimates of Abu Aqarib AFGs range from 0.21 to 2.32 kbar with an average of 1.33 kbar (Suppl. Table 2). These values correspond to emplacement depths of ~ 0.56 to 6.27 km (average ~ 3.58 km), assuming a crustal density of 2.7 g/cm^3^ (Suppl. Table 2). These conditions indicate shallow crustal emplacement^34,38^.

The calculated oxygen fugacity (ƒO₂) values (− 12 to − 9; Suppl. Table 2) indicate that Abu Aqarib AFGs were crystallized under reducing conditions. The relationship between log ƒO₂ and Tzr further suggests that crystallization occurred under conditions close to the Ni–NiO buffer (Suppl. Figure 1b), consistent with ferroan A-type granites^32^. Reduced conditions influence mineral stability and element distribution. They promote Fe enrichment in the melt and contribute to high FeO^T^/MgO ratios observed in the studied samples (Suppl. Table 2). Such redox affinity is commonly linked to low water activity and high-temperature magmatic systems^37^.

## Discussion

Sr–Nd–Hf–O isotopic and geochronological data are currently unavailable for the Abu Aqarib A-type granites. Consequently, the interpretations presented in this section regarding magma sources, emplacement timing, and hydrothermal fluid evolution rely primarily on integrated petrographic observations, mineral chemistry, and whole-rock geochemical evidence.

### Petrogenesis

#### Geochemical constraints on crust-mantle interaction

The petrogenesis of A-type granites remains debated, particularly regarding their source domains and the extent of mantle contribution. The Abu Aqarib hypersolvus AFGs exhibit geochemical characteristics indicating a dominant crustal origin together with features that may reflect limited mantle input.

Several geochemical features indicate a significant crustal contribution. The high SiO₂ contents, elevated differentiation indices (Suppl. Table 2), and metaluminous to slightly peraluminous character (Fig. 7a) are consistent with evolved felsic melts derived largely from continental crust. This interpretation is further supported by enrichment in incompatible elements such as Th, Rb, Pb, and ∑REEs (Suppl. Table 2), together with pronounced depletion in Sr, Ti, P, and Eu (Fig. 6b), which are typical characteristics of a strongly fractionated crust-derived granitic magmas^48^.

Further evidence for a crustal source is provided by the depletion of compatible elements and diagnostic trace-element ratios. The very low Cr and Ni contents (mostly below detection limits; Suppl. Table 2), together with low Mg# values (0.01–0.1; average ≈ 0.03), argue against PM-derived magmas, which are typically enriched in Cr and Ni and have higher Mg# (0.68–0.75)^49^. Likewise, the high Rb/Sr ratios (1.65–3.31; average = 2.66) and relatively low Ba/Rb values (~ 1.5–5.4; average = 4.03) are more consistent with crustal sources (Ba/Rb ≈ 6.72;^48^) than primitive mantle melts (Rb/Sr ≤ 0.1 and Ba/Rb ≈ 11;^50,51^). Moreover, the samples also plot close to the lower crust composition on the Nb/Y vs. Rb/Y diagram (Fig. 8b), further supporting a predominantly crustal source.

Although crustal melting appears to have been the dominant process, some geochemical features may indicate limited mantle involvement during magma evolution. These include enrichment in high field strength elements (HFSEs) (e.g., Nb, Zr, and Hf; Suppl. Table 2), the absence of pronounced negative Nb anomalies (Fig. 6b), and the lack of HREE depletion (~ 37–55 times chondritic values;^22^), features that have commonly been reported in A-type granites associated with mantle-derived thermal input^5,24,37^. In addition, Zr/Hf (36.21–44.73), Nb/Ta (~ 22.5–30), and Th/Nb (~ 0.09; Supp. Table 2) closely overlap with PM values (Zr/Hf ≈ 35–45, Nb/Ta > 18, and Th/Nb < 0.2;^50–54^) and differ from average continental crust compositions (Zr/Hf = 33, Nb/Ta ≈ 11‒12, and Th/Nb ≈ 0.4;^48,54–56^). Most samples also exhibit Y/Nb ratios < 1.2 (Suppl. Table 2), which have been interpreted as indicative of mantle-related signatures in A-type granites^38^. Their proximity to the mantle array (K/Rb = 1000) on the K₂O–Rb diagram (Suppl. Figure 1c) is likewise, consistent with this interpretation.

Nevertheless, these geochemical characteristics are not uniquely diagnostic of mantle input and may have alternative interpretations. For example, the elevated Nb contents and high Nb/Ta ratios of Abu Aqarib AFGs may result from equilibrium melting under ultrahigh-temperature (> 900 °C) conditions^57^ or by disequilibrium melting under high-temperature (~ 700–850 °C) conditions of purely crustal rocks, without any contribution from mantle sources^58^. Likewise, the lack of HREE depletion in Abu Aqarib AFGs can be explained by low-pressure melting of crustal rocks devoid of HREE-retentive minerals (e.g., garnet) in the source region, or by entrainment of peritectic garnet during melt segregation^59,60^. Moreover, trace-element concentrations and ratios in granitic magmas may be significantly modified during partial melting and fractional crystallization, causing them to deviate from primary source signatures. In addition, no coeval mafic or basaltic rocks have been documented in the Abu Aqarib area that would independently support a contribution from mantle-derived magmatism. Accordingly, although limited mantle involvement cannot be excluded, the available geochemical and geological evidence does not provide conclusive support for crust–mantle mixing.

Overall, the geochemical data indicate that the Abu Aqarib AFGs were derived predominantly from evolved continental crust, with geochemical signatures that may suggest a limited mantle contribution and/or mantle-derived thermal input during magma generation. This interpretation is consistent with the post-collisional extensional evolution of the ANS, where lithospheric relaxation may have facilitated asthenospheric upwelling and enhanced crustal anatexis. However, further isotopic investigations are required to evaluate the nature and extent of crust-mantle interaction.

#### Magma generation and evolution

The Abu Aqarib AFGs record a multistage magmatic evolution involving both melt generation and subsequent differentiation processes.

Partial melting played a primary role during magma generation. Geochemical characteristics, including reduced affinity (Fig. 8c), strongly fractionated REE patterns, enrichment in LREEs, and pronounced negative Eu anomalies (Fig. 6a), are all consistent with melting of metasedimentary sources under high-temperature conditions^7,10,36^. This inference is consistent with the source discrimination diagram (Fig. 8d), which indicates a significant metasedimentary contribution. The widespread occurrence of metasedimentary rocks in the Abu Aqarib area (Fig. 1b) provides additional geological support for this interpretation. Furthermore, the Th/Nd versus Th diagram (Suppl. Figure 1d) suggests that partial melting, rather than simple fractional crystallization, was the dominant process during magma generation^61^.

However, partial melting alone cannot account for the compositional diversity observed in the Abu Aqarib granites. The evolution of these magmas was controlled by fractional crystallization during magma ascent and emplacement. Pronounced depletion in Sr, P, and Ti observed in PM-normalized patterns (Fig. 6b) indicates extensive fractionation of feldspar, apatite, and Fe–Ti oxides, respectively^23^. The relatively high K_2_O contents in Abu Aqarib AFG, together with negative Eu anomalies (Suppl. Table 2), are consistent with extensive feldspar-controlled fractional crystallization^61^. Likewise, the negative correlation between Sr and Rb/Sr ratios (Suppl. Figure 1e) further supports progressive feldspar fractionation during magma evolution^62^. Moreover, the systematic relationship between Ba/Nb and SiO₂ (Fig. 8e) indicates continued K-feldspar fractionation during magma ascent and emplacement at shallow crustal levels.

In addition, assimilation–fractional crystallization (AFC) processes may have contributed to magma evolution. The negative correlation between Ce/Pb and SiO₂ (r = −0.7; Suppl. Figure 1f) suggests that crustal assimilation may have accompanied fractional crystallization during magma ascent. Because continental crust is generally enriched in Pb relative to Ce, interaction between the evolved felsic magma and surrounding crustal rocks may have contributed to the observed decrease in Ce/Pb ratios with increasing SiO₂. Possible assimilants include the Dokhan volcanic rocks and metasedimentary sequences that are widely exposed in the Abu Aqarib area. However, trace-element ratios in granitic magmas can also be modified by fractional crystallization processes^58^, making it difficult to distinguish definitely between crustal assimilation and magmatic differentiation using trace-element data alone. Therefore, although limited crustal assimilation cannot be excluded, the available geochemical data do not permit its extent or the nature of the assimilated component to be quantified. Moreover, the (La/Yb)_N_ versus La relationship (Fig. 8f) indicates that allanite fractionation played an important role in controlling REE distribution, particularly during the late stages of magmatic differentiation.

Consequently, the magmatic evolution of the Abu Aqarib AFGs was likely a multistage process. It began with partial melting of a metasedimentary-dominated lower crust under high-temperature, reduced conditions, possibly facilitated by mantle-derived thermal input during post-collisional extension. The resulting magmas subsequently evolved through extensive fractional crystallization processes involving both major mineral phases, particularly feldspars, and accessory minerals such as allanite, apatite, and Fe–Ti oxides, ultimately producing these highly evolved A-type granitic melts. Limited crustal assimilation may also have contributed to trace-element modification during magma evolution. Similar petrogenetic models involving crustal melting followed by extensive magmatic differentiation have been proposed for other A-type granites worldwide^63–66^, supporting the broader applicability of this interpretation.

### Geodynamic evolution

Abu Aqarib AFGs record a critical stage in the evolution of the ANS. Their geochemical signature (Figs. 7a, 8a) indicates emplacement during a post-collisional extensional regime. Similar tectono-magmatic models have been proposed for the northern ANS and other Neoproterozoic orogenic belts worldwide^4,67^.

**Fig. 9:**
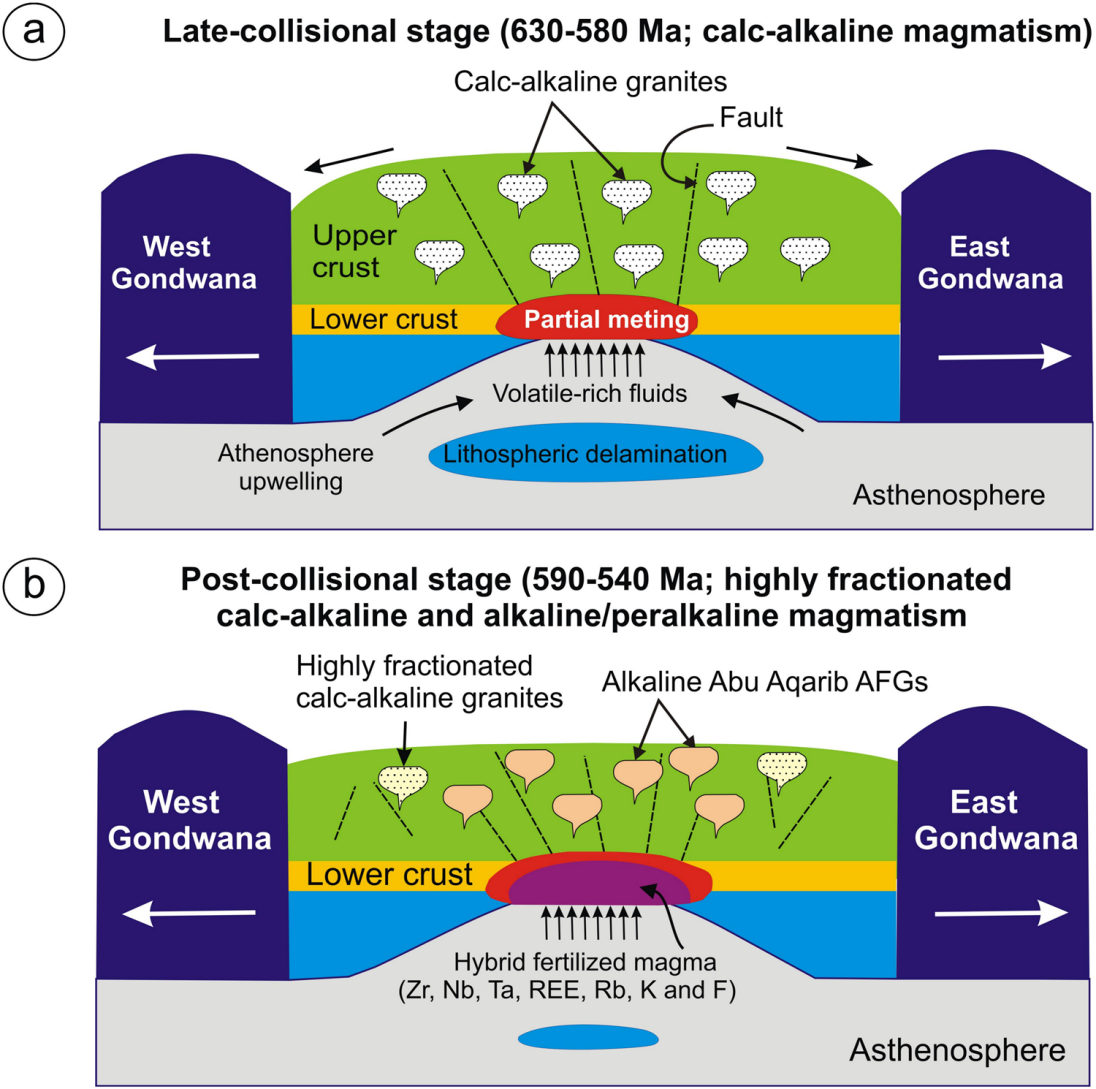
Conceptual geodynamic model illustrating a possible tectono-magmatic framework for the evolution of the Abu Aqarib AFGs within the ANS (modified after^69^ and^91^). (**a**) Regional late-collisional stage characterized by slab break-off and the onset of extensional collapse, likely associated with lithospheric delamination. (**b**) Regional post-collisional extensional stage involving asthenospheric upwelling and the circulation of volatile-bearing fluids (H₂O, CO₂, CH₄, F, B, and Cl), which may have promoted fluid–rock interaction and element redistribution. The A-type affinity of the Abu Aqarib granites, together with petrographic, mineralogical, and geochemical evidence for hydrothermal alteration and episyenitization, is based on the data presented in this study. In contrast, the tectonic processes shown in the model, including slab break-off, lithospheric delamination, asthenospheric upwelling, and their correlation with the late Neoproterozoic post-collisional evolution of the ANS, are inferred from previously published regional geodynamic models^69,89^. The temporal placement of these processes relative to the granite emplacement remains tentative and should be regarded as a preferred regional interpretation rather than direct evidence from the present study.

The evolution of the ANS began with continental collision between the juvenile ANS crust and West Gondwana (670–630 Ma), which caused significant crustal thickening during the early Neoproterozoic^1,68^. This was followed by slab break-off and subsequent lithospheric delamination during the late-collisional stage (~ 630‒580 Ma; Fig. 9a). These processes increased heat flow and triggered partial melting of the lower crust, generating calc-alkaline magmatism^4,68^.

During the post-collisional extension (~ 590–540 Ma), continued lithospheric thinning promoted asthenospheric upwelling^4,27,68^ (Fig. 9b). This enhanced thermal input into the crust and facilitated the generation of evolved felsic magmas (high-K calc-alkaline and alkaline/peralkaline magmas, Fig. 9b). Concurrently, volatile-rich fluids (e.g., H₂O, CO₂, F, and Cl) were released and circulated within the mantle lithosphere^69^. These fluids contributed to metasomatic modification of the lower crust and influenced melt composition (Fig. 9b). The Abu Aqarib alkaline AFGs were likely generated during this transitional stage through partial melting of felsic metasedimentary lower crust (Fig. 8a, d).

Structural controls also played a key role in magma ascent and emplacement. The NW–SE-trending Najd Fault System (620–540 Ma;^70^) acted as a major lithospheric-scale conduit for the upward migration of mantle-derived magmas, crustal melts, volatiles, and hydrothermal fluids throughout the ANS and may have facilitated emplacement of the Abu Aqarib AFGs (Fig. 9b).

Overall, the geochemical characteristics of the Abu Aqarib A-type granites are consistent with formation in a post-collisional setting within the ANS. Based on existing regional geodynamic models, their generation may have been associated with crustal extension, lithospheric thinning, and enhanced crustal melting during the late Neoproterozoic.

### Fluid-controlled metasomatism and rare-metal redistribution

Magmatic processes controlled the primary composition of the Abu Aqarib AFGs. However, late-stage fluid activity significantly modified their mineralogical and geochemical characteristics. Petrographic evidence, including quartz dissolution, vug development, and replacement textures (Fig. 3h, i) indicates extensive fluid–rock interaction and episyenitization. These features are consistent with the infiltration of Na-rich fluids along fractures and shear zones^71,72^. The presence of carbonate phases further suggests late-stage fluid evolution under changing physiochemical conditions, leading to carbonate precipitation within fractures, vugs, and dissolved quartz sites.

Mineral chemistry provides additional evidence for metasomatic modification. The pronounced Na enrichment and compositional variability observed in K-feldspar (Suppl. Table 1a) are consistent with alkali exchange during albitization. The nearly pure albite composition (Ab_93–99._) of plagioclase further indicates pervasive Na-metasomatism and extensive replacement of primary feldspar. The Fe-rich composition of chlorite reflects alteration of primary ferromagnesian minerals (amphibole and biotite) under low- to moderate-temperature hydrothermal conditions. In addition, replacement of magmatic magnetite by goethite records changes in redox conditions during fluid circulation. The incorporation of HREEs, F, Ca, and P in thorite also suggests interaction with F-bearing fluids, which likely played a key role in trace-element mobilization and redistribution.

**Fig. 10:**
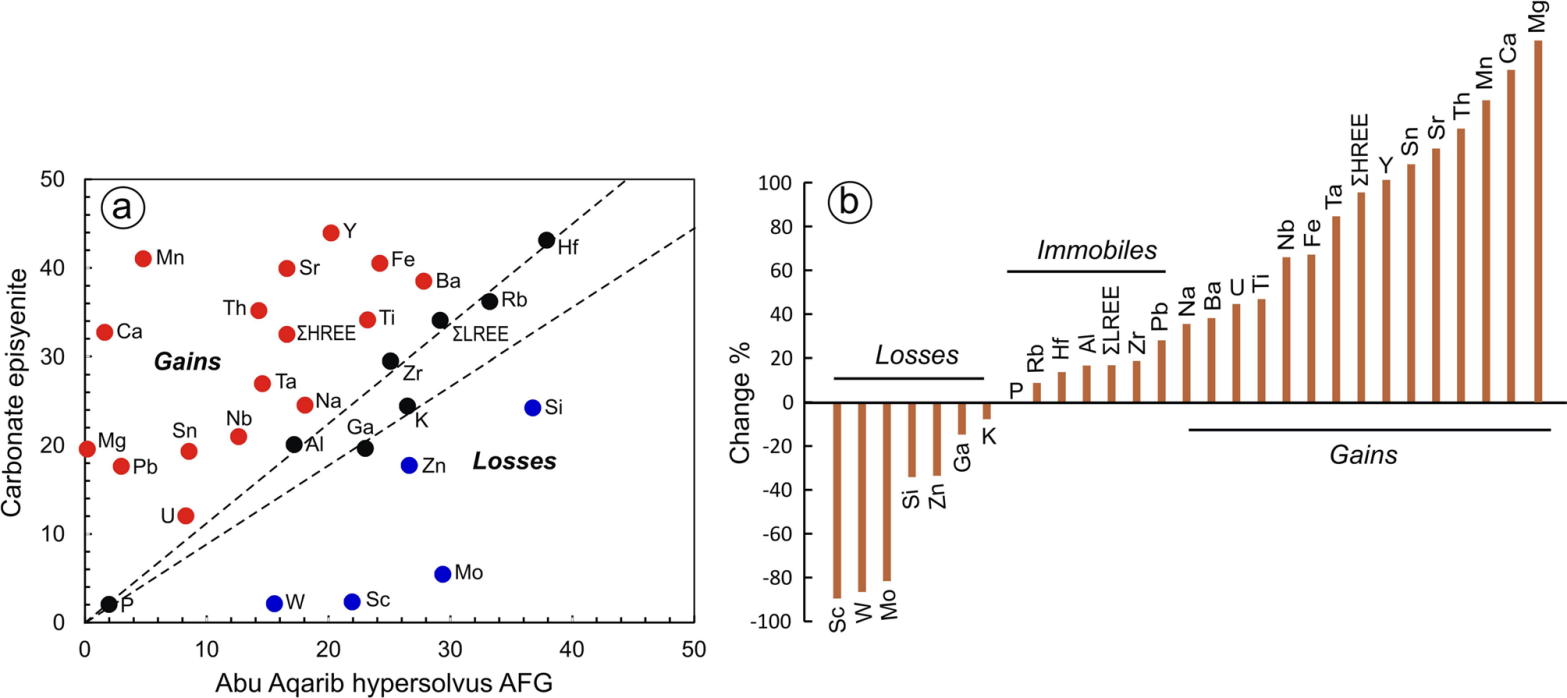
(**a**) Isocon diagram^73^, showing whole-rock chemical changes during episyenitization. The isocon lines are defined by relatively immobile elements and represents a gain/loss threshold of ± 20%. Elements plotting above and below the isocon lines indicate gains and losses, respectively, during metasomatic alteration. (**b**) Histogram showing percentage gains and losses of major and trace elements during alteration to episyenite. Major element data are in wt%, and trace elements are in ppm. All concentrations are scaled by random factors. Labels of major elements are abbreviated to their cation elements.

To evaluate elemental mobility during episyenitization, isocon analysis was applied to whole-rock geochemical data following the mass-balance approach of Grant^73^. This quantitative method compares concentrations of elements in altered and least altered samples and identifies immobile elements that define isocon lines. Deviations from these lines reflect relative mass gains or losses associated with fluid‒rock interaction. Elements plotting above the isocon lines indicate enrichment, whereas those plotting below them indicate depletion. The present isocons (Fig. 10a, b) are drawn, assuming no volume change during metasomatism, following previous studies^74–76^.

During the transformation of AFGs to carbonate episyenite (Fig. 10a), K_2_O, Al_2_O_3_, P_2_O_5_, Zr, Hf, Ga, and Rb remained immobile. In contrast, Si exhibited moderate loss (~ 35%, Fig. 10b), reflecting intense silica leaching during desilicification, whereas Na recorded a moderate increase (~ 36%), likely related to Na-metasomatism (albitization). This alteration was further characterized by substantial gain in Mg (~ 9200%, Fig. 10b), Ca (~ 1880%), Mn (~ 750%), Fe (~ 67%), and Ti (~ 47%), reflecting significant external fluid input and mineralogical re-equilibration. The marked enrichment in Ca and Mg, together with petrographic evidence of carbonate infillings and replacement textures, suggests precipitation of carbonate minerals from evolving late-stage hydrothermal fluids.

The isocon results also indicate substantial changes in the concentrations of several trace and rare-metal elements. Notable gains are observed in ∑HREEs (~ 96%), Sn (~ 125%), Th (~ 146%), U (~ 45%), Ta (~ 85%), Y (~ 117%), Nb (~ 66%), Ba (~ 38%), and Sr (~ 141%), whereas Sc (~ 90%), W (~ 87%), and Mo (~ 82%) show concomitant losses. These variations suggest that fluid–rock interaction promoted redistribution and local concentration of these elements during episyenitization. However, because the trace-element contents of the carbonate episyenite partly overlap those of the hypersolvus AFGs (Suppl. Table 2), the available data do not demonstrate systematic enrichment of all HFSEs and REEs at the deposit scale. Thorite represents an important host for HREEs (particularly Yb, Er, and Dy), whereas zircon largely preserves its primary HFSE signature, reflecting its resistance to post-magmatic alteration. Fe–Ti oxides may also accommodate minor amounts of HFSEs during subsolidus re-equilibration. Accordingly, episyenite formation represents an important mechanism for element redistribution and concentration during late-stage fluid–rock interaction rather than simply a product of alteration.

The relatively high F contents of both the hypersolvus AFGs (2293‒4430 ppm) and the carbonate episyenite (4037 ppm) suggest the involvement of F-bearing fluids during hydrothermal alteration. Fluorine may have facilitated the transport and redistribution of HFSEs and REEs during fluid-rock interaction under reduced conditions^77,78^. However, the available data do not demonstrate enrichment of fluorine in carbonate episyenite relative to the hypersolvus AFGs, and redistribution of fluorine from the original granitic system during metasomatism cannot be excluded.

The origin of the metasomatic fluids remains uncertain. Nevertheless, the close spatial association between the carbonate episyenite and the hypersolvus AFGs, together with petrographic and geochemical evidence for pervasive hydrothermal alteration, suggests that the episyenite most likely developed through fluid-mediated alteration of the hypersolvus AFGs. The metasomatic fluids were likely derived from late-stage exsolution of the evolving granitic magma; however, a contribution from externally sourced fluids cannot be excluded.

The available petrographic, mineralogical, and geochemical evidence supports a model involving: (1) generation of high-temperature, reduced A-type magmas predominantly from crustal sources, with possible limited mantle influence; (2) emplacement at shallow crustal levels in a post-collisional setting; (3) exsolution and circulation of hydrothermal fluids along structural pathways; (4) fluid–rock interaction leading to episyenitization; and (5) redistribution and local concentration of HFSE and REE. This sequence highlights the critical role of late-stage hydrothermal processes in modifying the primary geochemical characteristics of A-type granites. Therefore, the Abu Aqarib granites provide evidence that rare-metal redistribution in evolved granitic systems may result from the combined effects of magmatic differentiation and subsequent hydrothermal alteration. Similar processes have been documented in the ANS^79^ and other post-collisional granite provinces worldwide^71,72,80^.

## Conclusion

1. The Abu Aqarib granites are classified as hypersolvus alkali feldspar granites (AFGs) emplaced in the CED of Egypt. They exhibit typical A-type granite characteristics, including ferroan affinity, high SiO₂ and alkali contents, enrichment in HFSEs and REEs, pronounced negative Eu anomalies, high crystallization temperatures, and emplacement under shallow crustal and reduced conditions.
2. Geochemical data indicate that the Abu Aqarib AFGs were generated predominantly by partial melting of metasedimentary lower crust, with limited mantle contribution. Their evolution was controlled by magmatic differentiation processes within a geodynamic framework comparable to post-collisional extensional environments recognized elsewhere in the ANS. However, the temporal relationship between granite emplacement and specific regional tectonic processes remains to be verified through future geochronological investigations.
3. Late-stage magmatic–hydrothermal fluids extensively modified Abu Aqarib AFGs through episyenitization and carbonate precipitation. These processes promoted significant redistribution and local enrichment of HFSEs, REEs, U, Th, Nb, Ta, Sn, and Y, demonstrating that rare-metal concentration in the Abu Aqarib granites resulted from coupled magmatic and hydrothermal processes. Consequently, the Abu Aqarib granites represent a promising example of rare-metal enrichment associated with post-collisional A-type granite systems.

## Supplementary Information

Supplementary Information 1.

Supplementary Information 2.

Supplementary Information 3.

## Data Availability

All data generated or analysed during this study are included in this published article and its supplementary information files.
