# Supplementary material for: Crust‒mantle interaction and fluid evolution in the Arabian‒Nubian Shield: evidence from Abu Aqarib A-type granites, Eastern Desert of Egypt
Source: Sci Rep. 2026 Aug 18;16:25892. doi: 10.1038/s41598-026-65694-z (PMC13487301; doi:10.1038/s41598-026-65694-z)
Supplement: Supplementary file 1 [file 41598_2026_65694_MOESM2_ESM.pdf]

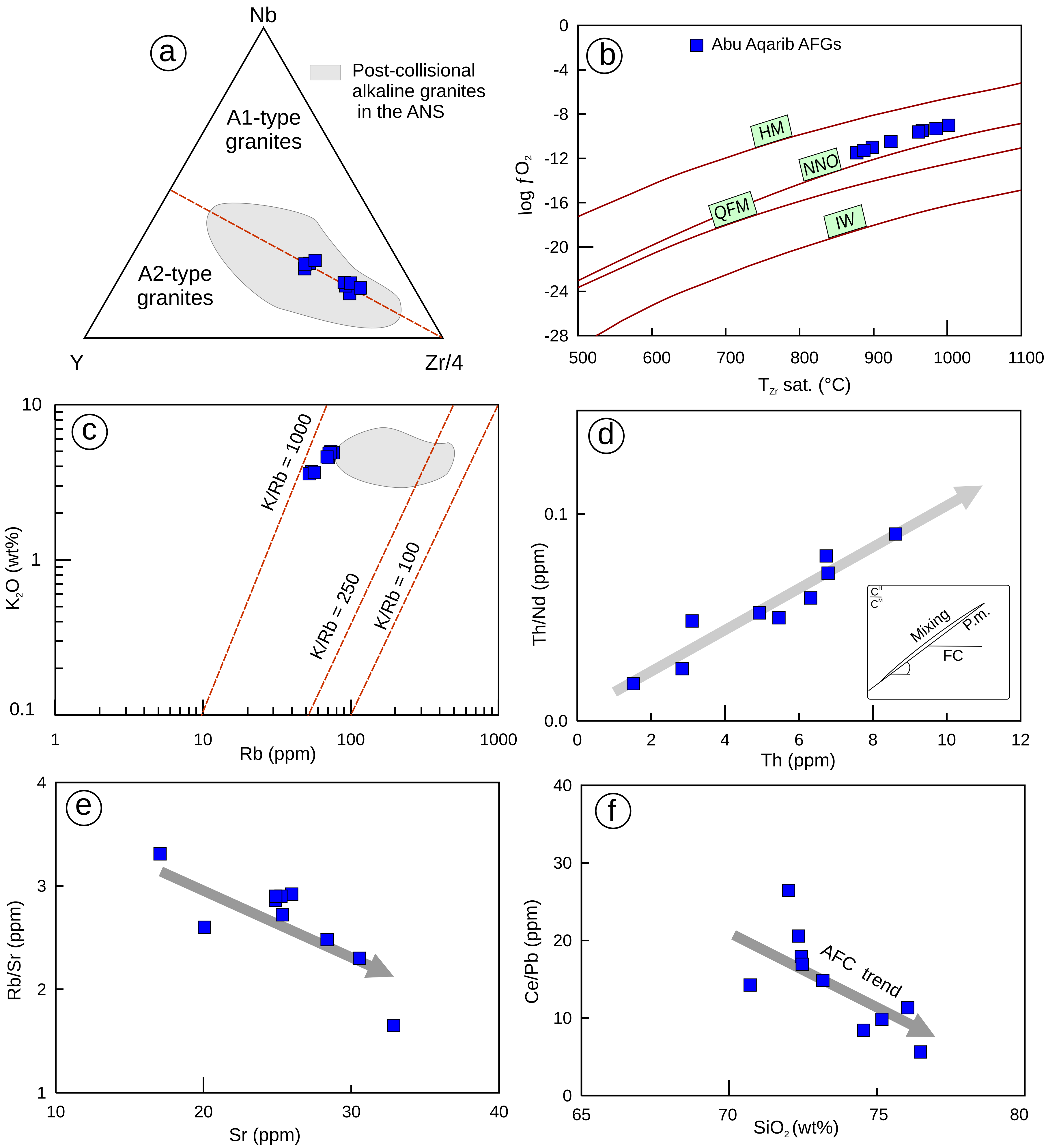

Suppl. Fig. 1. (a) Nb–Y–Zr/4 diagram (after Eby, 1992) to distinguish between A1 and A2 granites. **(b)** Plot of oxygen fugacity (fO_2) versus Zr-saturation temperature for the studied granites (after **Yang et al., 2020**). **(c)** K_2O vs. Rb diagram (after **Shaw, 1968**). **(d)** Th vs. Th/Nd binary diagram. Partial melting and fractional crystallization trends are from **Schiano et al. (2010)**. **(e)** Rb/Sr vs. Sr diagram (after **Singh and Vallinayagam, 2012**). **(f)** Ce/Pb vs. SiO_2 diagram. Assimilation-fractional crystallization (AFC) trend is from **Ayalew et al. (2019)**. Post-collisional alkaline/peralkaline granites in the ANS (**Katzir et al., 2007; El-Bialy and Streck, 2009; Azer, 2013; Khalil et al., 2018; Abd El Ghaffar and Ramadan, 2018**) are plotted for comparison.

Ayalew, D., Pik, R., Bellahsen, N., France, L. & Yirgu, G. Differential fractionation of rhyolites during the course of crustal extension, Western Afar (Ethiopian rift). G*eochem. Geophys. Geosyst.* **20**, 571–593 (2019).

Shaw, D.M. A review of K-Rb fractionation trends by covariance analysis. *Geochim. cosmochim. Acta* **32(6)**, 573-601(1968).

Singh, L.G. & Vallinayagam, G. Petrological and geochemical constraints in the origin and associated mineralization of A-type granite suite of the Dhiran Area, Northwestern Peninsular India. *Geosciences* **2(4)**, 66-80 (2012).

Yang, J.H. et al. Granite-related tin metallogenic events and key controlling factors in Peninsular Malaysia, Southeast Asia: New insights from cassiterite U-Pb dating and zircon geochemistry. *Econ. Geol.* **115(3)**, 581-601 (2020).
